# Poxvirus-based active immunotherapy synergizes with CTLA-4 blockade to increase survival in a murine tumor model by improving the magnitude and quality of cytotoxic T cells

**DOI:** 10.1007/s00262-016-1816-7

**Published:** 2016-03-10

**Authors:** Susan P. Foy, Stefanie J. Mandl, Tracy dela Cruz, Joseph J. Cote, Evan J. Gordon, Erica Trent, Alain Delcayre, James Breitmeyer, Alex Franzusoff, Ryan B. Rountree

**Affiliations:** Bavarian Nordic, Inc., 595 Penobscot Dr., Redwood City, CA 94063 USA; ExoThera LLC, 675 Olive Street, Menlo Park, CA 94025 USA

**Keywords:** Active immunotherapy, Poxvirus, Modified Vaccinia Ankara (MVA), Cancer, Anti-CTLA-4, Immune checkpoint blockade

## Abstract

The dramatic clinical benefit of immune checkpoint blockade for a fraction of cancer patients suggests the potential for further clinical benefit in a broader cancer patient population by combining immune checkpoint inhibitors with active immunotherapies. The anti-tumor efficacy of MVA-BN-HER2 poxvirus-based active immunotherapy alone or in combination with CTLA-4 checkpoint blockade was investigated in a therapeutic CT26-HER-2 lung metastasis mouse model. MVA-BN-HER2 immunotherapy significantly improved the median overall survival compared to untreated controls or CTLA-4 blockade alone (*p* < 0.001). Robust synergistic efficacy was achieved with the combination therapy (*p* < 0.01). Improved survival following MVA-BN-HER2 administration was accompanied by increased tumor infiltration by HER-2-specific cytotoxic T lymphocytes (CTL). These tumor-specific CTL had characteristics similar to antiviral CTL, including strong expression of activation markers and co-expression of IFNγ and TNFα. Combination with CTLA-4 blockade significantly increased the magnitude of HER-2-specific T cell responses, with a higher proportion co-expressing TNFα and/or IL-2 with IFNγ. Furthermore, in mice treated with MVA-BN-HER2 (alone or in combination with CTLA-4 blockade), the inducible T cell co-stimulator (ICOS) protein was expressed predominantly on CD4 and CD8 effector T cells but not on regulatory T cells (T_reg_). In contrast, mice left untreated or treated solely with CTLA-4 blockade harbored elevated ICOS^+^ T_reg_, a phenotype associated with highly suppressive activity. In conclusion, poxvirus-based active immunotherapy induced robust tumor infiltration by highly efficient effector T cells. Combination with CTLA-4 immune checkpoint blockade amplified this response resulting in synergistically improved efficacy. These hypothesis-generating data may help elucidate evidence of enhanced clinical benefit from combining CTLA-4 blockade with poxvirus-based active immunotherapy.

**Electronic supplementary material:**

The online version of this article (doi:10.1007/s00262-016-1816-7) contains supplementary material, which is available to authorized users.

## Introduction

The recent success of immune checkpoint inhibitors for the treatment of cancer has rejuvenated the field of cancer immunotherapy. Ipilimumab (Yervoy), a humanized monoclonal antibody specific for cytotoxic T lymphocyte-associated antigen-4 (CTLA-4), was the first FDA-approved drug from this new class of cancer immunotherapies. In patients with metastatic melanoma, ipilimumab treatment improved median overall survival (mOS) by 3.6 months and generated a complete response rate with durable tumor regression in 10–20 % of patients [1, 2]. Even greater clinical benefit may be achieved by selectively accelerating and augmenting anticancer immune responses through combination with active cancer immunotherapy.

PROSTVAC, a poxvirus-based active immunotherapy targeting the prostate-specific antigen (PSA), resulted in an 8.5-month increase in the mOS (25.1 vs. 16.6 months, *p* = 0.0061) in a randomized, placebo-controlled Phase 2 clinical trial of men with asymptomatic or minimally symptomatic castration-resistant metastatic prostate cancer (mCRPC) [3]. In a similar patient population, a Phase 1 trial of 30 subjects combined PROSTVAC immunotherapy with dose escalation of ipilimumab (1, 3, 5 and 10 mg/kg per dose), which resulted in a mOS of 31.3 months for all groups combined [4–6]. Patients who received PROSTVAC plus 10 mg/kg ipilimumab had a mOS of 37.2 months, and there appeared to be a tail on the survival curve with 20 % of patients alive at 80 months [6]. In addition, 58 % of chemotherapy-naïve subjects exhibited a PSA decline from baseline from the combination therapy [4].

Poxvirus-based active immunotherapies in additional cancer types are being investigated for clinical benefit as single-agent regimens, but clinical regimens exploring combinations with immune checkpoint inhibitors have not yet been initiated. For example, longer overall survival following treatment with CV-301, a poxvirus-based active immunotherapy which targets carcinoembryonic antigen (CEA) and mucin-1 (MUC-1), was observed compared to contemporary controls in metastatic colon cancer (*p* < 0.0001) [7]. In addition, MVA-BN-HER2, a recombinant modified vaccinia Ankara (MVA) vector expressing a modified form of the extracellular domain of human epidermal growth factor receptor 2 (HER-2), is under development for the treatment of breast cancer. MVA-BN-HER2 was well tolerated in two Phase 1 clinical trials and showed evidence of peripheral immune responses against HER-2 in more than two-thirds of subjects [8]. Evidence that anti-tumor efficacy is mediated by induction of HER-2-specific tumor-infiltrating lymphocytes (TILs) was generated in preclinical studies using MVA-BN-HER2 immunotherapy [9].

In this report, the impact of combining MVA-BN-HER2 active immunotherapy and CTLA-4 blockade on efficacy was evaluated in a therapeutic mouse tumor model. We then investigated the immune response profiles of peripheral and tumor-infiltrating T cells and identified characteristics that correlated with anti-tumor efficacy.

## Materials and methods

### Animals, tumor cell lines, and reagents

The CT26 murine colon carcinoma cell line expressing human HER-2 (CT26-HER-2) was licensed from the Regents of the University of California [10]; the genetic profile matched the established CT26 cell line (Idexx Radil, Columbia, MO, USA). A master cell bank and working cell banks were generated, and each bank was tested positive for HER-2 expression by flow cytometry (data not shown). Each bank was tested for a comprehensive list of pathogens including mycoplasma by Idexx Radil and was pathogen free. Cells were maintained and used as previously published [9].

Female BALB/c mice (6–8 weeks old, Simonsen Laboratories, Gilroy, CA) were implanted i.v. with 5.0 × 10^4^ CT26-HER-2 cells in 300 µL DPBS, which forms tumors in the lungs. On days 3 and 17 mice were treated with 200 µg of an antagonist anti-CTLA-4 antibody (Clone 9D9, Mouse IgG2b, Bio X Cell, West Lebanon, NH) by i.p. injection of 100 µL PBS. In studies to test for synergy and for the multicytokine functional assay, mice were treated with a dose titration of anti-CTLA-4 (200, 66, and 22 µg i.p. in 100 µL PBS) on days 3 and 17 or 4 and 18. On days 4 and 18 mice were treated with 1.0 × 10^7^ Inf.U MVA-BN-HER2 in 7.1 µL by tail scarification (t.s.). MVA-BN-HER2 (Bavarian Nordic, BN, Martinsried, Germany) is a modified vaccinia Ankara-based recombinant vector that encodes a modified form of the human epidermal growth factor receptor 2 (HER-2), referred to as HER2 [9]. The modified HER2 comprises the extracellular domains of HER-2 and contains two additional T helper epitopes to enhance immunogenicity [11]. The infectious unit titer (Inf.U/mL) of MVA-BN-HER2 was determined by flow cytometry [12]. Control mice were implanted with CT26-HER-2 cells on day 1 and received no additional treatment. Six months after the primary CT26-HER-2 challenge, mice that rejected tumors were implanted with 5.0 × 10^4^ CT26-WT cells in 300 µL DPBS.

To assess tumor burden, mice were euthanized on day 25 and lungs were weighed. In each group, 1–2 mice were euthanized and perfused with trypan blue (0.4 %, Mediatech, Inc., Manassas, VA) through the trachea; the lungs were collected, briefly submerged in hydrogen peroxide (30 %, EMD Millipore Corporation, Billerica, MA), washed in PBS, and photographed. For survival studies mice were euthanized at the first sign of distress. The BN Institutional Animal Care and Use Committee approved all animal procedures.

### Flow cytometry

Mice were treated as described above, and whole blood was collected via tail vein on days 10/11, 24/25 or 38. A strong T cell response was measured at day 24/25, 1 week after the second virus treatment. Subsequent studies examined tumor tissue at this time point when mice from each treatment as well as the control were alive. On day 24 or 25 whole blood was isolated by cardiac stick followed by collection of spleens and lungs for flow cytometric analysis. Tissues of 4–8 individual mice were analyzed for surface stains; tissues were pooled for 4–10 mice per group for the multicytokine and degranulation functional assays. Splenocytes were prepared by pressing the spleens between two frosted glass slides and lysing the red blood cells with ACK lysis buffer (Life Technologies, Grand Island, NY). Lungs and associated tumors were diced to ~1- to 2-mm^3^ pieces and further digested to single-cell suspensions for 1 h at 37 °C in DMEM with 10 % FBS, 50 U/mL DNAse I and 250 U/mL Collagenase I (Worthington Biochemical Corporation, Lakewood, NJ). Red blood cells in lungs and whole blood were lysed with RBC lysis buffer (eBioscience).

Antibodies against the following proteins were purchased from BD Bioscience (San Jose, CA): CD3e (500A2), CD4 (RM4-5), CD8a (53-6.7), CD11c (HL3), CD44 (IM7), CD62L (MEL-14), CD107a (1D4B), interferon-γ (IFN-γ) (XMG1.2), killer cell lectin-like receptor G1 (KLRG1, 2F1); from BioLegend (San Diego, CA): CD3e (145-2C11), CD127 (A7R34), IFNγ (XMG1.2), interleukin 2 (IL-2) (JES6-5H4), tumor necrosis factor-α (TNFα) (MP6-XT22); and from eBioscience (San Diego, CA): CD25 (PC61.5), ICOS (7E.17G9), CD127 (A7R34), FoxP3 (FJK-16 s), and CD16/CD32 (93).

To identify degranulating, cytotoxic T cells, single-cell suspensions of splenocytes (2 × 10^6^ cells/well) or tumor/lungs (1 × 10^6^ cells/well) were resuspended in RPMI-10 (10 % FBS, 1 % Pen-strep, and 0.1 % β-mercaptoethanol) and restimulated overnight at 37 °C in the presence of anti-CD107a antibody and Golgi Stop (BD Bioscience) [13]. The following peptides were synthesized by and purchased from JPT Peptide Technologies, Inc. (Acton, MA): MVA E3L and F2L (VGPSNSPTF and SPGAAGYDL, 1 µM each), HER-2 p63 (TYLPTNASL, 1 µM), HER-2 ECD overlapping peptide library (HER-2 OPL, 1 µM), and PSA (HPQKVTKFML, 1 µM) [9, 14–16]. Concanavalin A (ConA, MP Biomedicals, Santa Ana, CA) was used at 5 µg/mL as a positive control. The next day, cells were washed, blocked with anti-CD16/CD32 antibodies, and stained for surface markers. Cells were then washed, fixed/permeabilized with BD Cytofix/Cytoperm buffer, and stained intracellularly for IFNγ.

Additional intracellular cytokine staining was performed on splenocytes as described above except the anti-CD107a antibody was omitted, Golgi Stop and Golgi Plug were added, and cells were stained intracellularly for IFNγ, IL-2, and TNFα.

Regulatory T cells (T_regs_) were stained intracellularly for FoxP3 using the fixation/permeabilization buffers from eBioscience according to the manufacturer’s instructions.

All samples were acquired on the BD LSRII or Fortessa and analyzed using FlowJo version 9.6.2 (TreeStar Inc., Ashland, OR). For all flow cytometry graphs, the CD4^+^ and CD8^+^ T cells were gated on CD3^+^ lymphocytes.

### Statistical analysis

Statistical analyses were performed as described in the figure legends using GraphPad Prism version 6.01 for Windows (GraphPad Software, La Jolla, CA). For immunological data, data shown are the mean and standard error of the mean (SEM). An analysis of variance (ANOVA) with Holm–Sidak multiple comparisons test was used to determine statistical significance between treatment groups.

Synergy with the combination therapy in CT26-HER-2 challenged mice was determined by calculating the combination index (CI) using the Chou–Talalay method and CompuSyn software (www.combosyn.com) [17]. The method requires performing a dose titration of each therapy alone and in combination. The effect at each dose is defined as the fractional survival (fa) and is based on the mOS for each dose. Hundred days was considered long-term survival (fa ≈ 1). For example, with a mOS of 50 days, the fa = 0.5 (50 days/100 days). Multiple studies with the same treatment groups were pooled to determine the mOS. It was necessary to normalize the mOS with respect to the pooled control group to perform the analysis. CI < 1 indicates synergism, CI = 1 indicates additive effect, and CI > 1 indicates antagonism.

## Results

### MVA-BN-HER2 combined with an anti-CTLA-4 antibody eliminates tumors in an experimental lung metastasis model

**Fig. 1:**
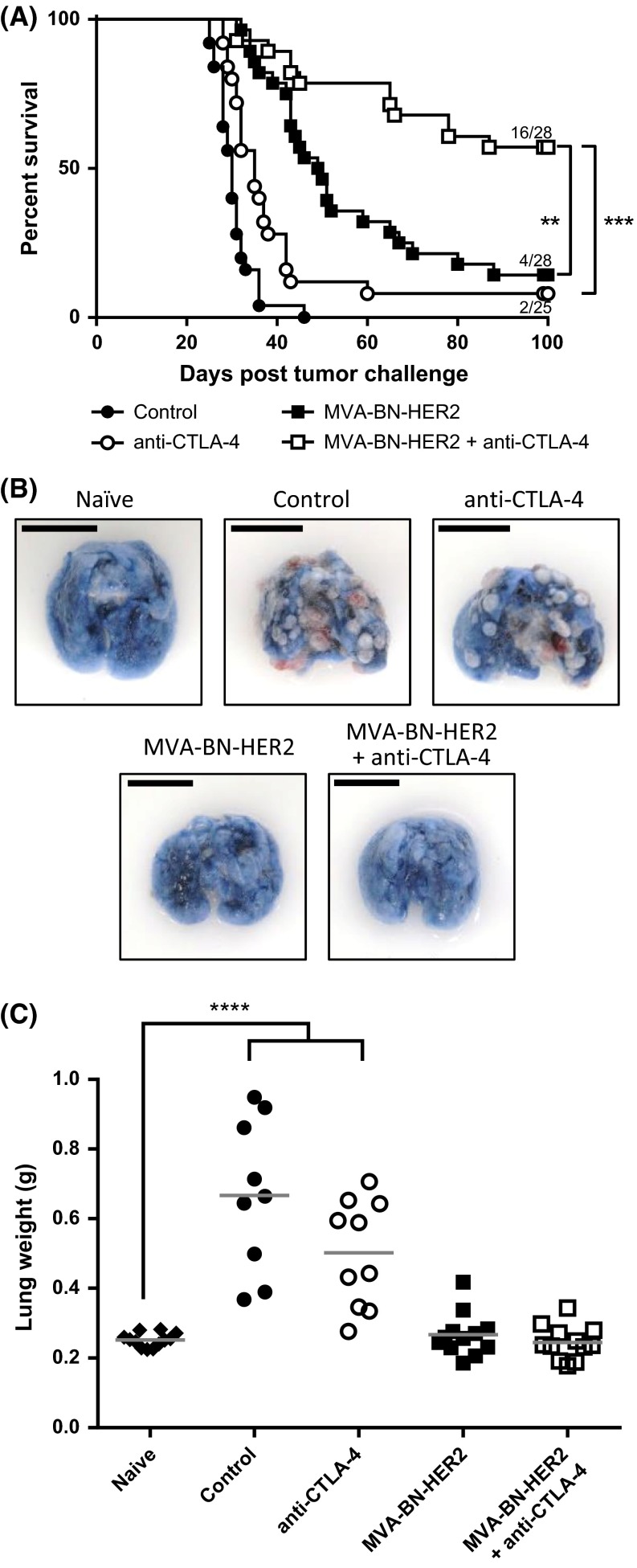
Overall survival and anti-tumor efficacy with MVA-BN-HER2 immunotherapy and CTLA-4 blockade. **a** Kaplan–Meier survival curves from the combined results of three independent studies. The median overall survival in control mice (30 days) was significantly extended with anti-CTLA-4 treatment (35 days, **p < 0.01), MVA-BN-HER2 treatment (49.5 days, ***p < 0.001), and MVA-BN-HER2+ anti-CTLA-4 combination therapy (>100 days, ***p < 0.001). A log-rank test with Bonferroni correction was used to determine significance between groups. **b** Representative lungs from each group harvested at day 25 were stained with Trypan Blue. Healthy lung tissue appears *blue*, while tumors appear *red* and *white*. *Scale bar* 1 cm. **c** Lung weights from treated mice on day 25 compared to naïve mice. A one-way ANOVA with Dunnett’s multiple comparisons test was used to determine significance between groups, ****p < 0.0001

To test whether the efficacy of MVA-BN-HER2 immunotherapy is augmented when combined with CTLA-4 immune checkpoint inhibition, mice were treated with MVA-BN-HER2 alone or in combination with an anti-CTLA-4 blocking antibody in a therapeutic HER-2^+^ experimental lung metastasis model. The combination of active immunotherapy plus checkpoint inhibition significantly and synergistically improved overall survival compared to either therapy alone [Fig. 1a, *p* < 0.001 vs. anti-CTLA-4 and *p* < 0.01 vs. MVA-BN-HER2; very strong synergy shown with a combination index (CI) < 0.001, see Supplemental Table 1]. The mOS of the combination therapy was greater than 100 days, significantly longer than either MVA-BN-HER2 treatment (49.5 days) or anti-CTLA-4 treatment (35 days) alone. In addition, more than half of the mice treated with the combination therapy (57 %) were still alive 100 days after tumor challenge, compared to 14 % of mice treated with MVA-BN-HER2 alone or 8 % of mice treated with anti-CTLA-4 alone. Next we tested the longevity of the induced anti-tumor response and evaluated whether the response had spread to antigens not encoded by the poxvirus-based active immunotherapy (antigen spreading). A small subset of mice that successfully rejected the initial CT26-HER-2 lung metastasis were rechallenged with CT26-WT cells (not expressing HER-2) 6 months after primary challenge. All rechallenged mice rejected the parental tumors, while naïve mice challenged with the same parental tumor cells had a mOS of 26 days (Supplemental Figure 1). These data demonstrate that long-lasting immune responses and antigen spread (expansion of the T cell repertoire from HER-2 to other cancer antigens) had occurred and were potent enough to protect mice from challenge with non-HER-2-expressing tumors.

Lungs of identically treated mice were removed 25 days after tumor implantation and assessed for tumor burden by lung weight. As seen in Fig. 1b, c, lungs of control mice or anti-CTLA-4 treated mice had numerous visible tumors at day 25, and the average lung weight was more than doubled compared to healthy naïve mice (*p* < 0.0001). In stark contrast, lungs of mice treated with MVA-BN-HER2 alone or in combination with anti-CTLA-4 appeared healthy with a similar appearance as the lungs of naïve unchallenged mice, and had no difference in lung weight (*p* > 0.99). These results highlight the benefit of MVA-BN-HER2 treatment to increase survival and reduce tumor burden.

### Poxvirus-based immunotherapy results in a distinct effector T cell phenotype

We hypothesized that phenotypic and functional analysis of the effector T cell responses at the predicted peak of treatment-induced activity, 1 week after the last treatment cycle, would reveal differences that later manifest as improved overall survival. Therefore, phenotypes of peripheral (spleen and blood) and tumor-/lung-infiltrating T cells from each treatment cohort were evaluated on day 25 by flow cytometry.

**Fig. 2:**
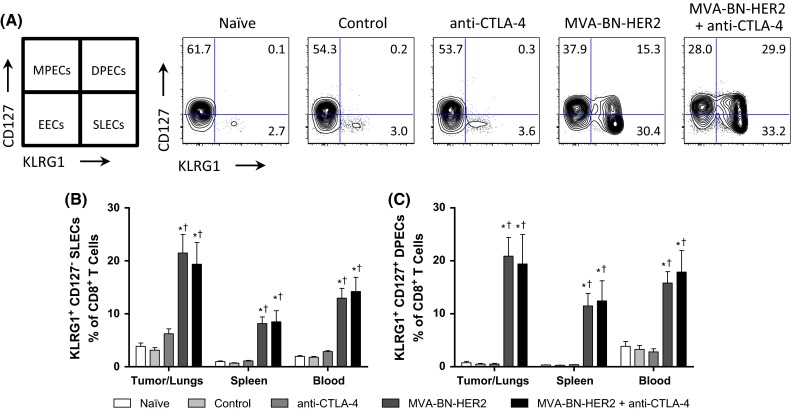
Effector T cell populations after MVA-BN-HER2 immunotherapy and/or CTLA-4 blockade. **a** Schematic for characterization of cells and a representative example of CD127 and KLRG1 expression in the tumor/lungs at day 25. **b** CD8^+^ CD127^−^ KLRG1^+^ short-lived effector cells (SLECs) and **c** CD8^+^ CD127^+^ KLRG1^+^ double-positive effector cells (DPECs). *MPECS* memory precursor effector cells, *EECs* early effector cells. *n* = 8 mice/group combined from two independent experiments. *p* < 0.05: * versus control, ^†^ versus anti-CTLA-4, and ^#^ versus MVA-BN-HER2

To determine whether there was a specific phenotype associated with MVA-BN-HER2 treatment that was augmented in combination with CTLA-4 blockade, we first looked at memory and activation markers induced by the different treatments. Effector memory cells generated in response to virus stimulation can be classified based on their expression of CD127 (IL7-Rα) and KLRG1. The KLRG1^−^ CD127^+^ subset is a long-lived memory precursor effector cell (MPEC) population, while the KLRG1^+^ CD127^−^ subset is known as short-lived effector cells (SLECs) [18]. The KLRG1^+^ CD127^+^ double-positive effector cells (DPECs) are believed to constitute a potent long-lived effector memory T cell subset [18, 19]. SLEC and DPEC CD8 T cell populations were detected in the lungs and peripheral tissue of mice following MVA-BN-HER2 administration, but not after anti-CTLA-4 treatment (Fig. 2a, b). Further analysis showed that the vast majority (>80 %) of both SLECS and DPECS were CD44hi CD62L^−^ (Supplemental Figure 2). The quality of the response induced by poxvirus-based immunotherapy was maintained by combination with anti-CTLA-4, but it was not augmented by combination therapy. Hence, the characterization of these SLEC and DPEC T cell populations at this time point was valuable for monitoring productive immunity in response to poxvirus-mediated immunotherapy.

### Combination therapy enhanced the population of tumor antigen-specific cytotoxic TILs

**Fig. 3:**
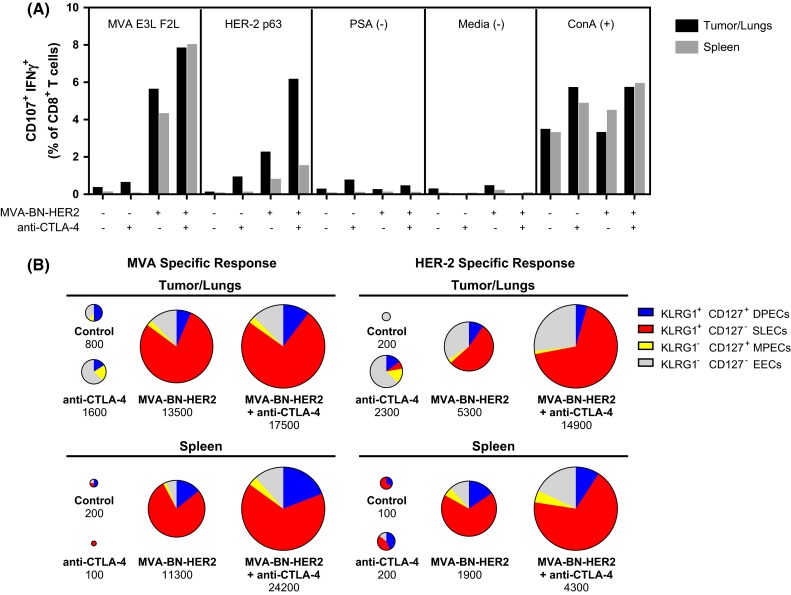
Degranulating T cells in the tumor/lungs or spleen of mice treated with MVA-BN-HER2 and/or CTLA-4 blockade. **a** Virus (MVA E3L F2L) and tumor antigen (HER-2 p63) specific responses were measured in the tumor/lungs and spleen; tissue was pooled from 4 mice/group. **b** Expression of KLRG1 and CD127 on the virus or HER-2 p63-degranulating (CD107a^+^ IFNγ^+^) cells from A. Pie charts are area-weighted to reflect the number of CD8^+^ CD107a^+^ IFNγ^+^ cells per million T cells

To determine whether the improved survival could be due to enhanced CTL function, antigen-specific cytotoxic T cell activity in the tumor/lungs and the periphery (spleen) was evaluated. MVA-BN-HER2 immunotherapy plus CTLA-4 checkpoint blockade led to a marked increase in the proportion of functional, HER-2-specific CD8 cytotoxic T cells infiltrating into tumor tissue (Fig. 3a). While MVA-BN-HER2 or anti-CTLA-4 therapy alone resulted in moderate induction of HER-2-specific CD8 TILs, there was no response in control mice. Of note, the HER-2-specific cytotoxic CD8 response was three- to fourfold higher in the tumor/lungs than in the spleen, while the virus-targeted response (i.e., stimulated by MVA-specific E3L and F2L peptides) alone or in combination with anti-CTLA-4 was similar in both tissues. Thus, HER-2-specific T cells preferentially homed to the tumor, and the magnitude of HER-2-specific CD8 TILs response correlated with the length of survival in the tumor model.

The degranulating cells that responded to either HER-2 p63 or MVA restimulation were predominantly SLECs (Fig. 3b), suggesting that the effector memory functions associated with the viral response phenotype also characterized cells responding to the HER-2 p63 antigen. Overall, anti-CTLA-4 monotherapy increased the cytotoxic CD8 TILs tenfold compared to mice that had received no treatment. However, MVA-BN-HER2 administration led to a 25-fold increase in numbers of HER-2-specific cytotoxic CD8 TILs compared to no treatment. This impact on HER-2-specific cytotoxic CD8 TILs was augmented to a 75-fold increase over controls following combination of active MVA-BN-HER2 immunotherapy with CTLA-4 checkpoint blockade.

### Combination therapy induces the expansion of polyfunctional CD8 T cells

**Fig. 4:**
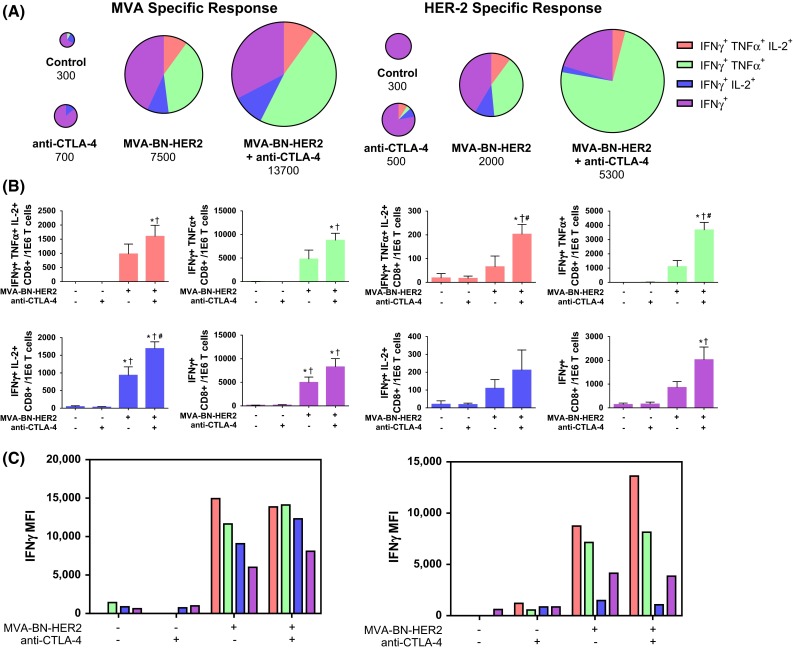
Antigen-specific cytokine production in CD8 T cells in the spleen. **a** Cytokine production in the spleen of mice treated with MVA-BN-HER2 and/or CTLA-4 blockade. Pie charts are area-weighted to reflect the number of CD8^+^ IFNγ^+^ cells per million T cells. **b** IFNγ, TNFα, and IL-2 subsets of cytokine^+^ T cells shown in **a**. **c** IFNγ MFI plotted for the subsets of cytokine^+^ T cells shown in A. Data combined from 5 experiments. In each experiment, splenocytes of 4–10 mice/group were pooled. *p* < 0.05: * versus control, ^†^ versus anti-CTLA-4, and ^#^ versus MVA-BN-HER2

The quality of the T cell response was further characterized by measuring IFNγ, TNFα, and IL-2 cytokine levels in stimulated splenic CD8^+^ T cells. In response to virus or HER2-p63 restimulation, a five- to tenfold increase in the magnitude of IFNγ^+^ T cells was found in mice treated with MVA-BN-HER2 compared to tumor-bearing mice that received no treatment (control) or CTLA-4 blockade alone, as shown by the relative size of the pie charts (Fig. 4a). The magnitude of the response to combination treatment was on average twofold larger as compared to the MVA-BN-HER2 treatment group after HER2-p63 restimulation (*p* < 0.01). The significant increase in IFNγ production with the combination therapy compared to MVA-BN-HER2 alone was observed only when splenocytes were restimulated with the tumor-specific antigen (HER2-p63) and not in response to restimulation with the poxvirus (MVA). Following MVA-BN-HER2 treatment, the expansion of IFNγ-producing antigen-specific cells was accompanied by a shift to a polyfunctional cytokine phenotype. For instance, CTLA-4 blockade alone induced CD8 T cells that were predominantly IFNγ single positive cells (depicted in purple). In contrast, more than 50 % of the IFNγ positive cells in MVA-BN-HER2-treated animals also produced TNFα (depicted in green) or IL-2 (depicted in blue), and a subset of those cells produced all three cytokines (depicted in orange). Combination treatment resulted in a statistically significant increase in this proportion of tumor antigen-specific (HER2-p63) cytokine-producing effector cells (Fig. 4b). A significantly higher percentage of the IFNγ^+^ TNFα^+^ IL-2^+^ or IFNγ^+^ TNFα^+^ polyfunctional HER-2 specific T cells were observed for the combination therapy as compared to MVA-BN-HER2 alone. This increase was specific for the HER-2 tumor antigen and was not observed in response to poxvirus-specific restimulation (MVA). Examination of the levels of IFNγ production from each of these CD8 T cell subsets was quantified by the mean fluorescence intensity (MFI) of each functional phenotype (Fig. 4c). On a per cell basis, polyfunctional cells produced more IFNγ than single positive cells. Overall, the cytokine profiles indicate that the functional quality of the tumor antigen-specific T cell response, in addition to the magnitude of the tumor-specific T cell response, is augmented even further by the combination of active immunotherapy plus CTLA-4 checkpoint blockade.

### Poxvirus-based active immunotherapy results in markedly improved T_eff_ to T_reg_ ratios within the tumor microenvironment

**Fig. 5:**
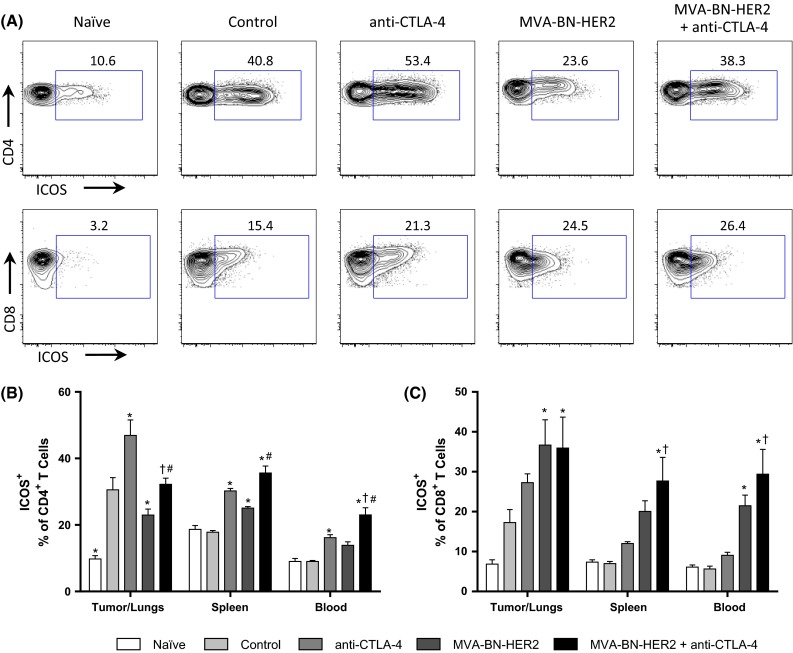
ICOS expression on CD4^+^ and CD8^+^ T cells. **a** Representative example of ICOS expression on CD4^+^ and CD8^+^ T cells in the tumor/lungs at day 25. **b** ICOS expression on CD4^+^ T cells in the tumor/lungs, spleen, and blood. **c** ICOS expression on CD8^+^ T cells. *n* = 8 mice/group combined from two independent experiments. *p* < 0.05: * versus control, ^†^ versus anti-CTLA-4, and ^#^ versus MVA-BN-HER2

The expression profiles of the inducible T cell co-stimulatory (ICOS) molecule were of particular interest because this T cell activation marker is known to increase on CD4^+^ T cells within the tumor and periphery following anti-CTLA-4 treatment [20]. CD4^+^ T cells exhibited elevated ICOS expression in the tumor/lungs of untreated controls and all treatment groups compared to naïve, tumor-free mice (Fig. 5a, b). Therefore, ICOS expression was induced on CD4^+^ T cells by the presence of the tumor without any treatment. Addition of the anti-CTLA-4 antibody alone raised ICOS expression to an even higher percentage of CD4^+^ T cells. Interestingly, ICOS levels were somewhat reduced on CD4^+^ T cells following MVA-BN-HER2 treatment compared to untreated controls, but were again higher when MVA-BN-HER2 was combined with CTLA-4 blockade. The increase in ICOS on CD4^+^ T cells was most prominent within the tumor/lungs, but elevated levels were also detected in the periphery of the treated groups but not in the control, tumor-bearing mice. Therefore, although differences in ICOS expression on CD4^+^ T cells were observed, they were not alone characteristic of protective anti-tumor immune responses.

Analysis of CD8 T cells revealed that in the tumor/lungs ICOS expression showed the highest increases in MVA-BN-HER2-treated mice irrespective of CTLA-4 treatment (Fig. 5c). In the blood and spleen, ICOS expression was consistently elevated on CD8^+^ T cells of mice treated with MVA-BN-HER2 in combination with anti-CTLA-4, compared to control or anti-CTLA-4 treated mice. Thus, while the percentage of ICOS^+^ CD8^+^ T cells was significantly higher in the periphery with combination treatment, the responses observed in the tumor were similar in MVA-BN-HER-2-treated groups irrespective of anti-CTLA-4 treatment. This demonstrates that the poxvirus-based immunotherapy drives the quality of the induced immune response.

**Fig. 6:**
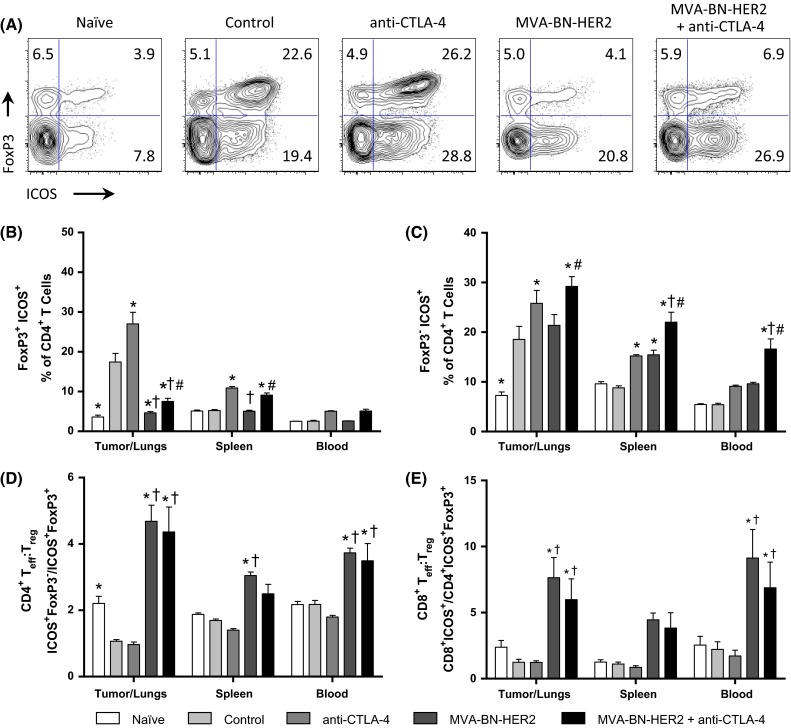
ICOS expression on effector and regulatory T cells. **a** Representative example of ICOS and FoxP3 expression in the tumor/lungs at day 25. **b** ICOS expression on FoxP3^+^ *T*_regs_. **c** ICOS expression on FoxP3^−^ CD4 T cells. **d** CD4 ICOS^+^ *T*_eff_ to ICOS^+^ *T*_reg_ ratio (CD4^+^ ICOS^+^ FoxP3^−^/CD4^+^ ICOS^+^ FoxP3^+^) and **e** CD8 ICOS^+^ *T*_eff_ to ICOS^+^ *T*_reg_ ratio (CD8^+^ ICOS^+^/CD4^+^ ICOS^+^ FoxP3^+^). *n* = 8 mice/group combined from two independent experiments. *p* < 0.05: * versus control, ^†^ versus anti-CTLA-4, and ^#^ versus MVA-BN-HER2

Elevated ICOS expression on CD4^+^ T cells characterizes the activation of effector cells (T_eff_) as well as highly suppressive T_regs_ [21, 22]. We therefore examined which class of CD4^+^ T cells expressed ICOS in the different treatment groups (Fig. 6a). ICOS was elevated on FoxP3^−^ T_eff_ cells but not T_regs_ following MVA-BN-HER2 treatment (Fig. 6b, c), and ICOS was even more pronounced on FoxP3^−^ T_eff_ cells following combination therapy (Fig. 6c). This resulted in a marked increase in the ratio of both CD4^+^ and CD8^+^ ICOS^+^ T_eff_ to ICOS^+^ T_regs_ in the tumor/lungs and periphery in mice receiving MVA-BN-HER2 treatment compared to control mice (Fig. 6d, e). In contrast, in tumor-bearing control mice and mice treated only with anti-CTLA-4 where tumor burden was high, ICOS expression was increased on both FoxP3^+^ T_regs_ and FoxP3^−^ T_eff_ cells (Fig. 6b, c). Overall, the high ICOS^+^ T_eff_ to ICOS^+^ T_reg_ ratio induced by MVA-BN-HER2 alone or in combination with CTLA-4 blockade likely reflects a more effective immune response and correlated with strong anti-tumor efficacy.

## Discussion

We found that combining the poxvirus-based active immunotherapy with CTLA-4 checkpoint blockade strongly synergized to increase overall survival in a therapeutic mouse tumor model. In this combination, the poxvirus-based active immunotherapy drove the quality of the response, which was characterized by highly activated polyfunctional T cells; this T cell phenotype was further amplified by CTLA-4 treatment.

Treatment with MVA-BN-HER2, but not anti-CTLA-4 alone, led to a dramatic increase of KLRG1 expression on CD8 T cells which were categorized with CD127/IL-7Rα expression as short-lived effector cells (SLECs, KLRG1^+^ CD127^−^) or an effector-like memory subset of double-positive effector cells (DPECs, KLRG1^+^ CD127^+^) [19]. Induction of SLECs and DPECs was driven by the poxvirus-based immunotherapy and was not further expanded in the combination therapy group. The SLEC and DPEC phenotypes were also identified in mice treated with PROSTVAC and MVA poxvirus-based active immunotherapies [23, 24]. Interestingly, the SLEC and DPEC populations described here expanded for both virus-specific and HER-2-specific degranulating T cells. This indicates that the effector characteristics associated with the viral response also defined the T cells responding to the encoded HER-2 tumor-associated antigen. It may therefore be possible to identify poxvirus-induced T cells responding to the inserted tumor antigens in patients treated with poxvirus-based active immunotherapy by including virally induced markers in phenotyping panels [25].

The quality of the T cell responses is often assessed by the profile of cytokines produced upon activation, with co-expression of multiple cytokines defining more active effector cells [26]. Examination of a multicytokine profile of IFNγ, TNFα, and IL-2 revealed that poxvirus immunotherapy promoted co-expression of cytokines on CD8 T cells. This expansion in polyfunctional T cells was present in response to both the virus and the tumor antigen. Combination treatment with CTLA-4 blockade significantly increased the number of IFNγ^+^ TNFα^+^ IL-2^+^ triple-positive and IFNγ^+^ TNFα^+^ double-positive cells specific for the HER-2 tumor antigen but not the poxvirus vector (MVA), and this correlated with increased mOS. Furthermore, the magnitude of HER-2-specific cytotoxic activity was increased in TILs of combination-treated animals.

ICOS is expressed on activated T cells as a positive co-stimulatory molecule, and its expression on peripheral T cells may provide a biomarker for therapeutic benefit with anti-CTLA-4 therapy [20, 27–29]. Our results revealed that treatment with CTLA-4 blockade augmented the number of ICOS^+^ CD4^+^ T cells but not ICOS^+^ CD8^+^ T cells in the tumor, blood, and spleen. In contrast, MVA-BN-HER2 treatment was generally accompanied by an expansion of ICOS^+^ CD8^+^ T cells, but this population was not further increased with CTLA-4 blockade. Though ICOS expression alone did not reveal the additional benefit of combining poxvirus immunotherapy with checkpoint inhibition, these data suggest that higher expression of ICOS on effector cell populations may correlate with improved survival.

Importantly, poxvirus-based immunotherapy, alone or in combination with anti-CTLA-4 blockade, led to an increase in the ICOS^+^ T_eff_ to ICOS^+^ T_reg_ ratio. This shift in the ICOS^+^ T_eff_ to ICOS^+^ T_reg_ ratio was most pronounced at the tumor site, but it was also apparent in the spleen and blood. Unlike earlier reports, the T_eff_/T_reg_ ratio did not increase following CTLA-4 inhibition alone [30–32], even though there was a modest increase in overall survival (5 days). This may be due to a couple of factors. First, when the ICOS^+^ T_eff_ to ICOS^+^ T_reg_ ratio was measured mice receiving CTLA-4 blockade alone had a large tumor burden, thus the shift in ICOS^+^ T_eff_ to ICOS^+^ T_reg_ populations may no longer have been apparent. Second, the isotype of the anti-CTLA-4 antibody is critical for the T_reg_ depleting activity of the anti-CTLA-4 antibody [33]. The IgG2a isotype, but not the IgG2b isotype of the anti-CTLA-4 antibody used in the present study, led to selective reduction of intratumoral T_regs_ [33]. Previous studies have shown that combining MVA-BN-HER2 with T_reg_ depletion further increased the mOS compared to MVA-BN-HER2 alone [9]. Therefore, combining MVA-BN-HER2 with an anti-CTLA-4 antibody with T_reg_ depleting properties may lead to an even higher ICOS^+^ T_eff_ to ICOS^+^ T_reg_ ratio and could potentially further enhance the efficacy of the combination therapy.

Antigen spread is the development of novel immune responses against target antigens expressed by the patient’s own tumor. Antigen spread responses are thought to be a crucial component of successful immunotherapy as they expand the anticancer immune repertoire beyond the initially targeted antigen. Rechallenge experiments with non-HER-2-expressing tumors demonstrated that the immune responses were durable and were accompanied by antigen spread to additional tumor antigens. These data corroborate previous preclinical findings targeting HER-2, PSA, or CEA [9, 23, 34]. More importantly, antigen spread responses were also detected in clinical studies in 68 % of patients treated with PROSTVAC [35]. Immune responses to additional tumor antigens included PSMA (prostate-specific membrane antigen), PAP (prostatic acid phosphatase), MUC-1, and Brachyury [4, 36].

The present study was performed in mice where human HER-2 is not a self-antigen, and immune responses could be lower in a tolerant model. Anti-tumor efficacy and immune responses were demonstrated in transgenic mice expressing human CEA as a self-antigen using CEA-targeted poxvirus-based active immunotherapy [37, 38]. Furthermore, when these poxvirus-based immunotherapy candidates were used in combination with CTLA-4 blockade, increased lymphocyte proliferation, IFNγ production, and T cell avidity resulted in tumor rejection through immune responses to self-antigens in tolerant mouse models [39–41]. Our present study further characterized the effect of combination therapy by investigating the magnitude and quality of TILs and peripheral lymphocytes. Our examination of viral and tumor antigen-specific responses revealed that the effector T cell characteristics associated with the viral response also defined the T cells responding to the encoded HER-2 tumor antigen and were further augmented in combination with CTLA-4 blockade. Thus, the preclinical data generated here provide hypothesis-generating evidence for elucidating the potential correlates of improved T cell effector responses resulting from combination poxvirus-based immunotherapy plus CTLA-4 checkpoint blockade.

In the clinic, monotherapy with PROSTVAC, a PSA-targeted poxvirus-based active immunotherapy, significantly extended the mOS from 16.6 to 25.1 months in patients with metastatic castration-resistant prostate cancer [3]. Ipilimumab alone provided a nonsignificant 1.2-month improvement in overall survival compared to the placebo group in a Phase 3 trial of patients with prostate cancer [42]. Combining PROSTVAC with CTLA-4 blockade in a similar patient population resulted in an encouraging mOS of 31.3 months in a Phase 1 study of fixed dose PROSTVAC with a dose escalation of Ipilimumab [3, 5, 6]. Of note, 20 % of patients treated with the highest dose of ipilimumab were still alive at 80 months [6]. Importantly, immune-related adverse events commonly associated with immune checkpoint inhibition were not exacerbated by combination with PROSTVAC immunotherapy [4]. Thus, the data from this work elucidating potential immunologic correlates for improved anti-tumor response in preclinical models together with a suggestion of improved overall survival in the clinic support further clinical investigation of combination poxvirus-based active immunotherapy together with CTLA-4 immune checkpoint inhibition in prostate cancer and other cancers.

## Conclusion

In summary, we showed that combination of poxvirus-based active immunotherapy with CTLA-4 blockade resulted in synergistic anti-tumor efficacy. Functional and phenotypic characteristics of the immune response were driven by the poxvirus-based active immunotherapy and the antigen-specific effector T cell responses were further amplified by CTLA-4 blockade. These data provide evidence that may translate into the potential immunologic correlates of productive anticancer immunity and clinical benefit resulting from poxvirus-based active immunotherapy combined with CTLA-4 immune checkpoint inhibition.

## Electronic supplementary material

Below is the link to the electronic supplementary material.

Supplementary material 1 (PDF 271 kb)

## Abbreviations

CEA: Carcinoembryonic antigen
CI: Combination index
CTL: Cytotoxic T lymphocytes
CTLA-4: Cytotoxic T lymphocyte-associated antigen-4
CV-301: Poxvirus-based active immunotherapy targeting CEA and MUC-1
DPECs: Double-positive effector cells
EECs: Early effector cells
HER-2: Human epidermal growth factor receptor 2
HER2: Modified extracellular domain of HER-2 and two additional T helper epitopes
ICOS: Inducible T cell co-stimulator
IFN: Interferon
IL: Interleukin
KLRG1: Killer cell lectin-like receptor G1
mCRPC: Metastatic castration-resistant prostate cancer
MFI: Mean fluorescence intensity
mOS: Median overall survival
MPECs: Memory precursor effector cells
MUC-1: Mucin-1
MVA: Modified vaccinia Ankara
MVA-BN-HER2: Poxvirus-based active immunotherapy targeting HER-2
PAP: Prostatic acid phosphatase
PROSTVAC: Poxvirus-based active immunotherapy targeting PSA
PSMA: Prostate-specific membrane antigen
PSA: Prostate-specific antigen
SLECs: Short-lived effector cells
T_eff_: Effector T cells
TILs: Tumor-infiltrating lymphocytes
TNF: Tumor necrosis factor
T_regs_: Regulatory T cells
