# Supplementary material for: Poxvirus-based active immunotherapy synergizes with CTLA-4 blockade to increase survival in a murine tumor model by improving the magnitude and quality of cytotoxic T cells
Source: Cancer Immunol Immunother. 2016 Mar 10;65(5):537–49. doi: 10.1007/s00262-016-1816-7 (PMC4840227; doi:10.1007/s00262-016-1816-7)
Supplement: Supplementary file 1 — Supplementary material 1 (PDF 271 kb) [file 262_2016_1816_MOESM1_ESM.pdf]

Supplemental Table 1 Combination of MVA-BN-HER2 and anti-CTLA-4 immunotherapies led to synergy in a mouse lung metastasis model

**MVA-BN-HER2 (Inf. U)**	**anti-CTLA-4 (mg/kg)**	**mOS (Days)**	**Combination Index (CI)**	**Description**
0	0	29	N/A	N/A
1E5	0	30	N/A	N/A
1E6	0	32	N/A	N/A
1E7	0	46	N/A	N/A
0	1	31	N/A	N/A
0	3	30	N/A	N/A
0	10	33	N/A	N/A
1E7	1	51	0.149	Strong Synergism
1E7	3	60	0.013	Very Strong Synergism
1E7	10	84	<0.001	Very Strong Synergism

Mice were implanted i.v. with CT26-HER-2 cells on day 1 and treated with MVA-BN-HER2 (t.s., days 4 & 18) or anti-CTLA-4 (i.p., Clone 9D9 on days 3 &17 or 4 &18) at doses indicated in the table. Note that survival studies reported in the main body of the paper were treated with anti-CTLA-4 on days 3 & 17 only, therefore the mOS values reported in the paper and Supplemental Table 1 are similar but not identical.

Description and Interpretation of CI from Chou TC:

**CI**	**Description**
<0.1	Very Strong Synergism
0.1 – 0.3	Strong Synergism
0.3 – 0.7	Synergism
0.7 – 0.9	Moderate Synergism
0.9-1.10	Nearly Additive
1.10-1.45	Moderate Antagonism
1.45-3.3	Antagonism
2.2 – 10	Strong Antagonism
>10	Very Strong Antagonism

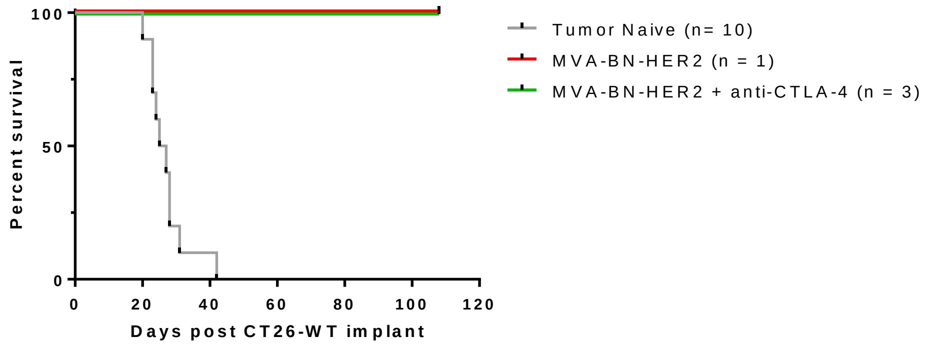

Supplemental Figure 1 Durable immune response with antigen spread after tumor rejection. Six months after the primary CT26-HER-2 challenge, mice that rejected tumors were implanted with CT26-WT cells. In the same experiment, CT26-WT cells were implanted into control (tumor naïve) mice that were not previously challenged with CT26-HER-2 cells.

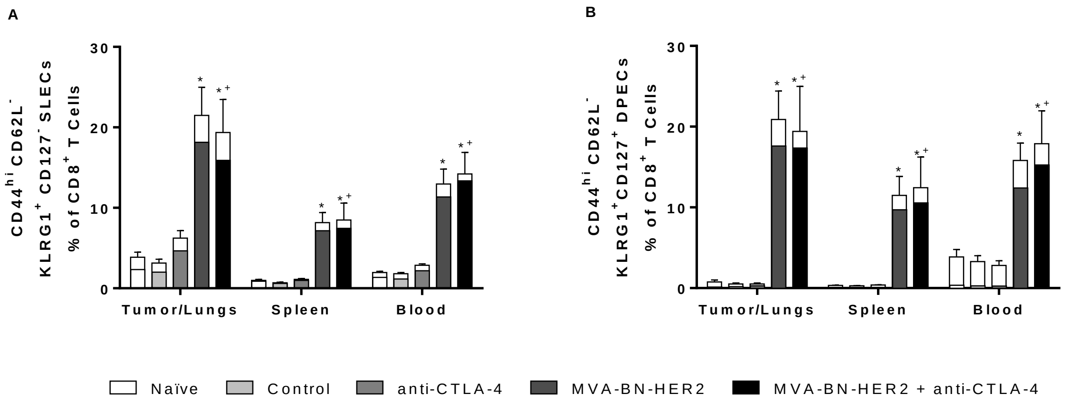

Supplemental Figure 2 MVA-BN-HER2 alone or in combination with CTLA-4 blockade increased the effector cell population. Cells were defined by their expression of CD127 and KLRG1 (empty bars) and the effector memory markers CD44^{hi} and CD62L^{-} (shaded bars). A) CD8^{+} CD127^{-}KLRG1^{+} short lived effector cells (SLECs) and B) CD8^{+} CD127^{+}KLRG1^{+} double positive effector cells (DPECs). $p < 0.05$: * vs. control, † vs. anti-CTLA-4, and # vs. MVA-BN-HER2.
